# The Clinical and Microbiological Effects of LANAP Compared to Scaling and Root Planing Alone in the Management of Periodontal Conditions

**DOI:** 10.3390/diagnostics13142450

**Published:** 2023-07-22

**Authors:** Edwin Sever Bechir

**Affiliations:** Faculty of Dental Medicine, George Emil Palade University of Medicine, Pharmacy, Science, and Technology of Targu Mures, 38 Gh. Marinescu Str., 540142 Targu Mures, Romania; edwin.bechir@umfst.ro

**Keywords:** SRP, LANAP, standard PCR-PET test

## Abstract

The purpose of this study was to evaluate the efficiency of two therapeutic procedures clinically and microbiologically in the management of periodontally affected teeth: scaling and root planing alone and the laser-assisted new attachment procedure (LANAP). Molecular biological determinations of bacterial markers through the polymerase chain reaction (real-time PCR) method with standard PET tests (species-specific DNA probes at a time) were used for the quantification of three of the most important periodontal pathogens (*Aggregatibacter actinomycetemcomitans*, *Porphyromonas gingivalis*, and *Treponema denticola*). Both nonsurgical periodontal therapies were proven effective in patients with chronic periodontal disease; however, LANAP was associated with a greater reduction in pocket depth and improved clinical outcomes, associated with a significant decrease in the amount of *Porphyromonas gingivalis*. The clinical results included a decrease in periodontal pocket depth, bleeding on probing, and dental plaque, with LANAP having better overall outcomes than SRP alone. The use of Nd:YAG lasers in LANAP therapy is a safe and effective procedure that is well accepted by patients.

## 1. Introduction

Periodontal disease is a complex and multifactorial inflammatory condition of an infectious nature, in which the supporting tissues of the teeth (gingival mucosa, periodontal ligaments, and alveolar bone) are involved, and it leads to the loss of periodontal attachment [1,2]. Periodontal disease, characterised by the destruction of connective tissue and alveolar bone tissue, induces an immune and anti-inflammatory response of the human body to pathogens [3]. Currently, periodontal disease is considered the most common chronic bacterial infectious disease among humans, which is why it represents an essential concern for global public health [4]. The determining role in the progression of periodontal disease is played by oral cavity hygiene through the microbial factor, and the other favouring or predisposing factors [5,6]. The factors that influence periodontal disease include the rate of dental plaque deposition, dental calculus, dental caries, dental migrations, edentation, occlusal trauma, dento-maxillary anomalies, the existence of parafunctional habits, bad habits, local iatrogenic factors, and the existence of systemic factors and circumstantial factors favouring the occurrence of this disease [6,7,8,9].

The classification of periodontal and peri-implant diseases in 2017 had considerable consequences for dental practice. The terms “aggressive” and “chronic” periodontitis were replaced with a single category, “periodontitis”. The classification, after staging and grading, promotes a dimensional periodontal diagnosis, which offers the clinician the possibility of obtaining an individualised diagnosis and treatment plan for every patient. The severity and extent of the disease are based on the measurable degree of destroyed and damaged tissue [10]. The development of periodontitis is categorised into stages I to IV, while the disease complexity is classified into Grades A, B, or C [11].

From a microbiological point of view, periodontal disease is characterised by quantitative and qualitative changes in the oral microbiome of the subgingival area of the affected teeth [12,13]. The determining factors, unanimously accepted by specialist researchers engaged in the etiopathology of periodontal disease, are represented by both microbial virulence and the implications of the modified immune response against this virulence, which ultimately lead to the irreversible destruction of the periodontium [6,14]. In conformity with the expanded Human Oral Microbiome Database (eHOMD), there are 774 oral bacterial species, 8% of which are officially named, 16% are unnamed but cultivated, and 26% are uncultivated phylotypes [15]. Gram-negative anaerobic bacterial species such as *Aggregatibacter actinomycetemcomitans*, *Porphyromonas gingivalis*, *Treponema denticola*, *Tannerella forsythia*, *Prevotella intermedia*, *Peptostreptococcus micos*, and *Fusobacterium nucleatum* are predominant in moderate and deep periodontal pockets, determining the progression of periodontal disease and the irreversible damage of the periodontium [16,17,18,19].

The main objectives in the treatment of periodontal diseases are the elimination of inflammation and the prevention of additional bone loss, followed by the periodic controls necessary to assess the evolution of periodontal disease [2,6]. The evolution of periodontal therapies has witnessed multiple nonsurgical and surgical options and techniques [20,21]. The majority of the forms of periodontitis can be addressed with nonsurgical therapy, which involves dental plaque control, scaling, and root planing (SRP), and the results can be maintained through long-term monitoring [21,22].

Today’s laser technologies are utilised in many dental specialties, like periodontology, paediatrics, oral surgery, implantology, and oral pathology [23,24,25].

Different types of dental lasers, with wavelengths between 400 and 10,600 nm, have been studied to enhance the performance of periodontal treatments [26,27,28,29,30]. Many studies have evaluated clinical and microbiological responses to periodontal treatments. The clinical efficiency of the Nd:YAG laser was demonstrated by the positive results obtained in the ablation of potentially haemorrhagic granulation tissue, obtaining a thick coat of coagulated tissue, in addition to hemostasis [29,31,32].

The LANAP (laser-assisted new attachment procedure) utilises a laser beam for the disintegration of pathogenic bacteria and the elimination of the damaged periodontal tissues through the sterilisation of the compromised areas, promoting tissue regeneration without surgery, regenerating the damaged periodontal tissues and developing an attachment to the root surfaces of the newly formed connective tissues, but the Nd:YAG laser alone cannot remove dental calculus in difficult areas [33,34].

Some studies have indicated that, in patients with fixed prosthetic restorations, abutment teeth are more prone to periodontal inflammation. The fact that age, the duration of use of the fixed prosthetic restoration, and the marginal adaptation of the prosthetic elements affect periodontal health are reasons why periodontal therapy can often be required for these teeth, with minimal or noninvasive means such as laser therapy [35,36].

This study aimed to comparatively evaluate the clinical and microbiological outcomes of scaling and root planing alone and LANAP in periodontally affected teeth that required rehabilitation using fixed prosthetic restorations.

## 2. Materials and Methods

### 2.1. Participants

This research was accomplished with the implementation of the ethical principles of the Declaration of Helsinki and good clinical practice. The research protocol was authorised by the Ethics Committee of “George Emil Palade” University of Medicine, Pharmacy, Science, and Technology of Târgu Mureș, Romania (number 750 from 18 February 2020).

All selected patients were notified regarding the requirements of the study, and only those who willingly accepted the requirements of the research program were admitted. The stages of the study and the necessity for monitoring were explained to each selected subject. Before the start of the study, written informed consent was obtained from all participants regarding the use of their data and samples for scientific purposes.

The study was conducted in the Integrated Dental Center of Dental Medicine Faculty of “George Emil Palade” University of Medicine, Pharmacy, Science, and Technology of Târgu Mureș and Dentaltop Dentistry Clinic in Târgu Mureș. The research period was between February 2020 and October 2022, with an intermission due to the context of the COVID-19 pandemic.

A thorough anamnesis, accurate clinical examinations, and orthopantomography X-ray examinations were carried out for all patients to assess eligibility. The anamnesis consisted of questions regarding name, age, address, employment, the existence/absence of any allergies, nutritional habits, smoking, parafunctional habits/bruxism/vicious habits, and acute and/or chronic illnesses. A single examiner carried out oral examinations to avoid calibration errors. They consisted of the inspection of the soft and hard tissues of the oral cavity, oral hygiene assessment, and positioning and degree of periodontal disease. The probing depths, clinical attachment loss, visible plaque index, bleeding on the probing index, and the mobility degree of the teeth included in the study were recorded in the periodontal chart. The periodontal chart can record tooth mobility, furcation involvement, gingival margin (mm), probing depth (mm), and notes regarding the presence or absence of plaque and bleeding on probing on six sites per tooth. Complementary radiographic examinations were carried out for all patients, and their orthopantomograms were assessed.

The inclusion criteria in the study consisted of men and women of at least 18 years of age who agreed to participate in the study and signed the patient’s informed consent form; patients with at least twelve natural teeth present, distributed in the four quadrants; patients presenting at least two periodontally affected teeth, each presenting periodontal pockets with a depth of at least 4 mm on at least one of the six dental surfaces examined during probing; patients suffering from at least stage II, grade B, localised periodontitis; patients smoking less than ten cigarettes per day; patients presenting fixed prosthetic restorations or subsequently requiring this type of oral rehabilitation; and patients with clinically and radiologically evidenced bone resorption.

The exclusion criteria involved patients who have undergone periodontal treatment within the last 12 months; patients with systemic/local antibiotic therapy within the last 6 months; patients suffering from stage I or stage II, grade A, localised periodontitis; patients smoking more than ten cigarettes per day; systemic conditions that can change the therapeutic result (type 1 and 2 diabetes, immune deficiencies, HBV, HCV, cancer, haematological disorders, and epilepsy); pregnancy and breastfeeding; teeth with an indication of extraction; inability or refusal to follow the study protocol.

All included patients were selected in conformity with the same study criteria and were confirmed with at least two teeth with more than 4 mm periodontal pocket depth in different quadrants. The randomisation of the teeth was computer-generated (Random.org service).

Of the 15 selected patients (30 teeth included in the study), 1 patient withdrew. The remaining 14 participant patients (28 teeth), 7 females and 7 males, were aged between 36 and 67 (the average age of patients being 51.5 ± 15.5 years).

**Figure 1:**
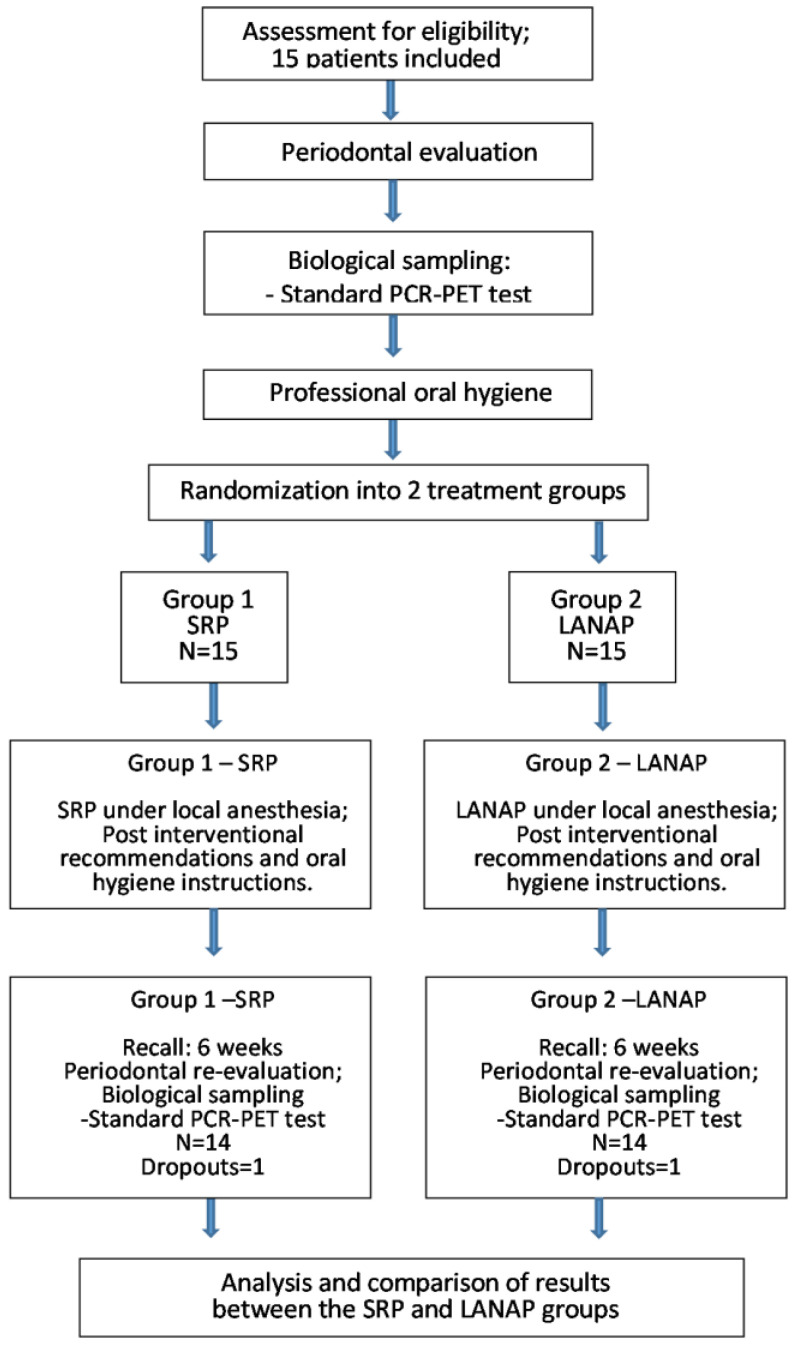
Flow diagram of the study protocol.

The clinical protocol applied in the study comprised the following procedures (Figure 1):

- Specialist consultation, assessment of eligibility, the recording of periodontal parameters in a periodontal chart, and complementary radiographic examinations (OPGs) were carried out;
- Patients were informed of the stage of the condition at the time of presentation;
- Patients with at least stage II, grade B, localised periodontitis, and periodontal pocket depths of at least 4 mm, were selected according to the inclusion and exclusion criteria;
- The selected patients were informed about the requirements of the study and the implementation of the treatment plan, and informed consent was obtained;
- The first biological sampling was carried out using a standard PCR-PET test;
- Professional dental cleaning was then performed;
- The selected teeth of the patients were randomly divided into two groups;
- SRP was performed on the first group of teeth included in the study and in the remaining quadrants that were not included in the study;
- The LANAP protocol was applied to the second group of teeth;
- Patients underwent training on the implementation of correct dental hygiene procedures at home;
- All patients received postinterventional instructions and the recommendation to use chlorhexidine mouthwash twice a day for two weeks after performing brushing at home. The Bass brushing technique was recommended, with soft bristle manual brushes and fluoride toothpaste;
- A new recording of dental and periodontal status and a second standard PCR-PET test for both groups of teeth included in the study were performed after six weeks.

### 2.2. Clinical Evaluation and Treatment

The diagnosis of periodontal tissue status was carried out by examining the visible plaque index, bleeding on the probing index, and the degree of periodontal involvement.

The clinical parameters were recorded in the periodontal chart at baseline. Based on the examination, 2 test teeth (from different quadrants of each patient) with at least stage II, grade B, localised periodontitis; periodontal pocket depths ≥ 4 mm; and bleeding on probing were selected for testing, resulting in 28 teeth. The site with the deepest probing depth was selected for microbiological sampling for each tooth.

**Figure 2:**
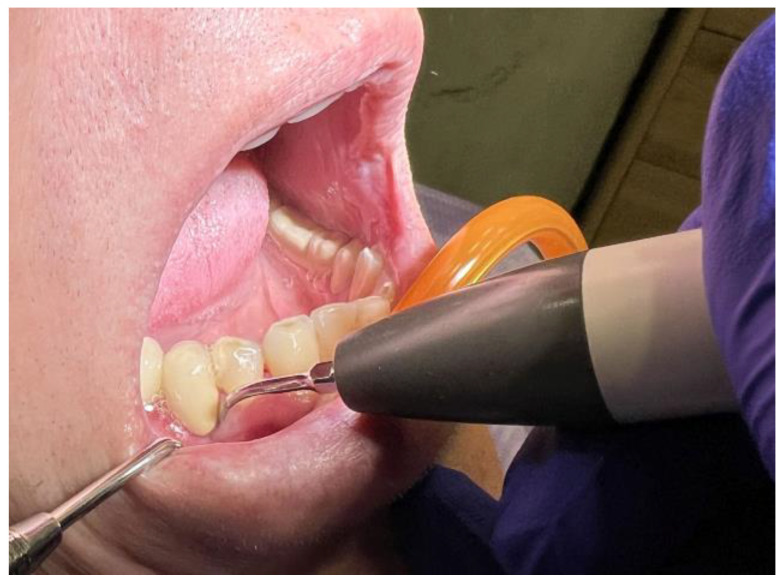
Ultrasonic scaling.

Professional dental cleaning was performed with the Satelec Newtron ultrasonic scaler device (Acteon^®^ Group, Jersey City, NJ, USA) for all the patients included in the study to remove calculus deposits (Figure 2), followed by the polishing of the tooth crowns surfaces with rotating brushes, prophylactic paste, and airflow, using the Air-N-Go Airflow (Acteon^®^ Group, Jersey City, NJ, USA).

Two quadrants of each patient were randomly assigned to one of the two treatment groups as follows: Group 1: SRP as monotherapy (scaling and root planing) 1 week after ultrasonic scaling, professional brushing, and airflow; Group 2: LANAP (the application of the Nd:YAG laser in the periodontal pockets, scaling and root planing, followed by a second application of the Nd:YAG laser) 1 week after ultrasonic scaling, professional brushing, and airflow.

**Figure 3:**
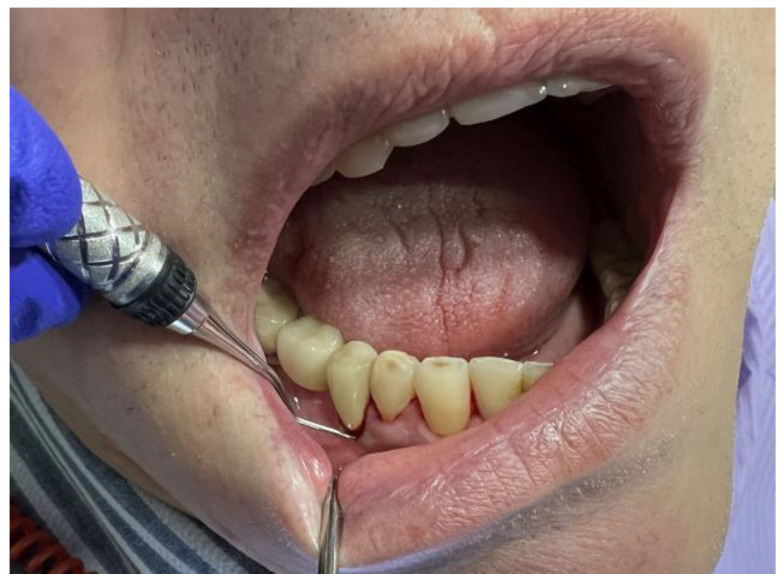
Gracey curette used for manual SRP.

SRP was performed under local anaesthesia on the teeth included in the first group and on the two remaining quadrants that were not included in the study one week after the microbiological sampling, ultrasonic scaling, professional brushing, and airflow. Area-specific Gracey curettes (HuFriedy^®^ Group, Chicago, IL, USA) were used for manual scaling and root planing (Figure 3). SRP was performed until the root surfaces became smooth, and there was no visual or tactile evidence of calculus or altered root cementum.

LANAP was performed using the Nd:YAG laser 1064 nm (Lightwalker AT-S, Fotona^®^, Ljubljana, Slovenia). The first application of the Nd:YAG laser (1064 nm) was performed under local anaesthesia with a power setting of 2.5 W, MSP pulse (duration of 100 µs and 20 Hz) for 20 s per each tooth. The objective of placing the laser’s optical fibre in the gingival sulcus is to remove the damaged epithelium of the periodontal pockets. The optical fibre (0.3 mm) was placed parallel to the long axis of the tooth, and lateral and apical movements were performed. The fibre was apically inserted 1 mm less than the measured periodontal pocket depth.

**Figure 4:**
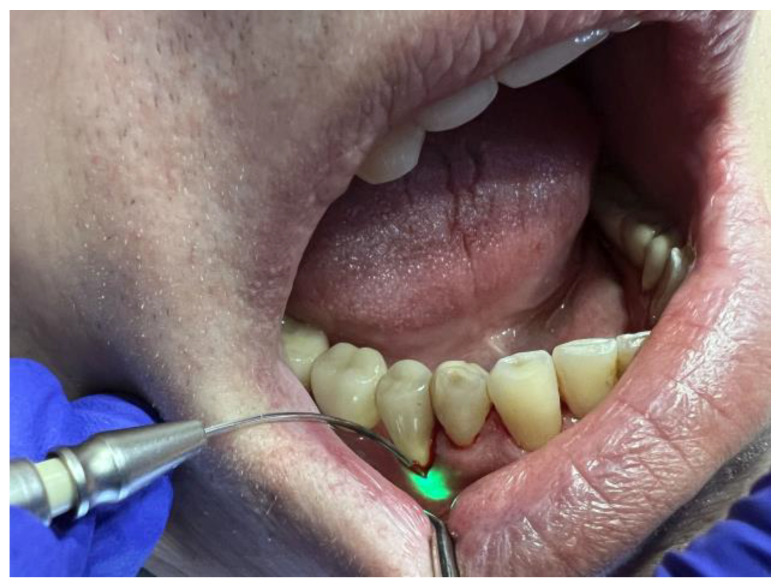
LANAP application.

After thorough scaling and root planing, the fibre optic system of the Nd:YAG laser was applied for a second time inside the periodontal pockets, with a power setting of 3.5 W and VLP pulse (duration of 600 µs and 20 Hz), for 20 s per tooth, aiming to obtain a fibrin clot and seal the periodontal pocket (Figure 4). All clinical procedures were conducted according to minimally invasive therapy procedures.

The training of the patients included educating and motivating them to correctly implement oral hygiene procedures at home twice a day. In addition to oral hygiene instructions, the patients were instructed to use a chlorhexidine solution 0.12% twice a day for two weeks (Curasept ADS^®^, Curaden AG, Kriens, Switzerland) after the interventions as part of plaque control.

Six weeks after the applied therapies were completed, the oral cavity health status was assessed, the dental and periodontal status was once again recorded in the periodontal chart, and a second microbiological standard PCR-PET test (MIP Pharma GmbH, Blieskastel-Niederwürzbach, Germany) was conducted on the studied teeth.

### 2.3. Microbiological Assessment

**Figure 5:**
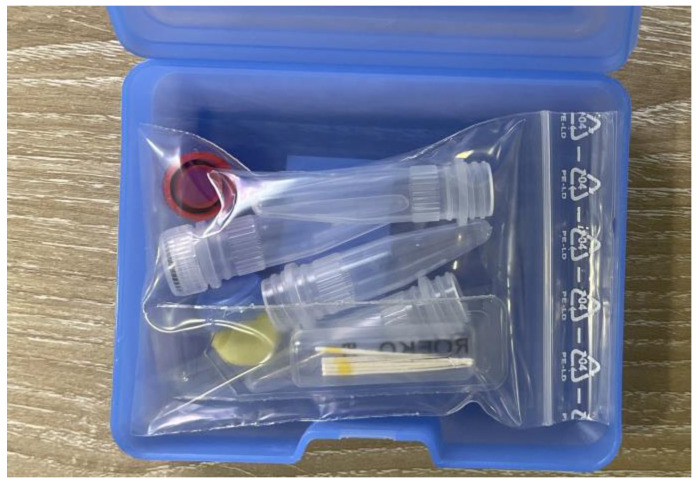
Content of PET-DiagnosticSet box: instructions, transfer tubes, paper cones.

The microbiological assessment for every patient was carried out using the standard PCR-PET test kit (MIP Pharma GmbH, Blieskastel-Niederwürzbach, Germany), which includes the instructions, the transfer tubes, the paper cones, and the patient information sheet (Figure 5).

**Figure 6:**
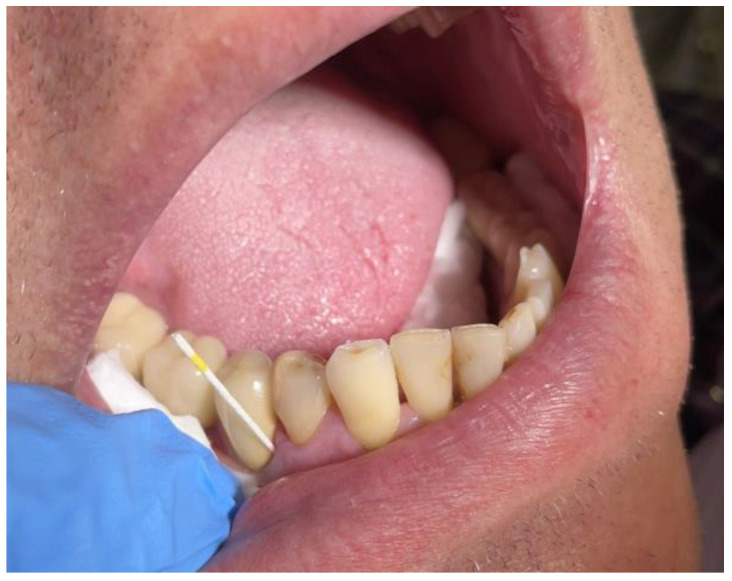
Microbiological sample collection.

The bacterial load was assessed before and after the treatments in the two groups of teeth. Before the professional dental hygiene, the first assessment was accomplished in the first session for the teeth included in both study groups. The first sampling was mandatory before the institution of any treatment and was carried out at the level of the tested sites with the help of the paper cones available in the collection kit. Two samples were collected from periodontal pockets belonging to different quadrants. The sampling sites were isolated and dried, and the supragingival plaque was removed. The paper cones were inserted into the periodontal pockets up to their base with the help of sterile dental tweezers (Figure 6). The samples were collected from periodontal pockets with depths that exceeded 4 mm.

**Figure 7:**
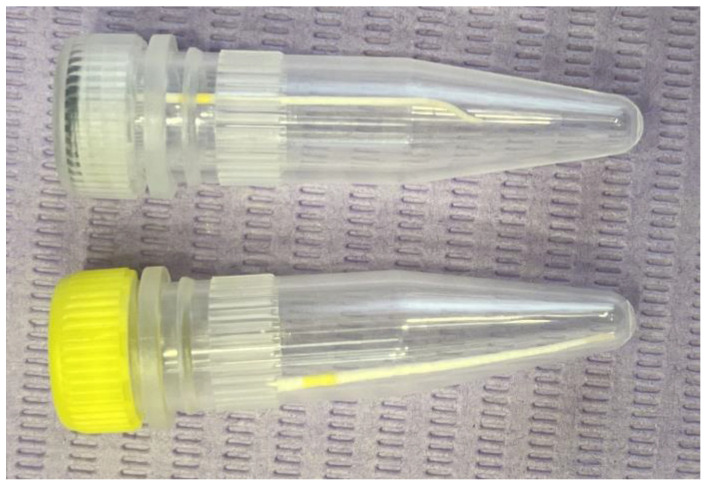
PET-DiagnosticSet: collected microbiological samples inside the tubes.

The cones were held inside the periodontal pocket for 20 s and then withdrawn. Contact with saliva or the epithelium of the oral cavity was avoided. The cones were inserted into transfer tubes and placed in the collection kit with a sheet containing the patient’s information (Figure 7).

The standard PCR-PET tests were conducted at the MIP Pharma laboratory, with the aim of qualitatively and quantitatively determining three periodontal pathogens from the collected samples, namely *Aggregatibacter actinomycetemcomitans* (*Aa*), *Porphyromonas gingivalis* (*Pg*), and *Treponema denticola* (*Td*), microorganisms that are risk indicators of severe types of periodontitis.

**Figure 8:**
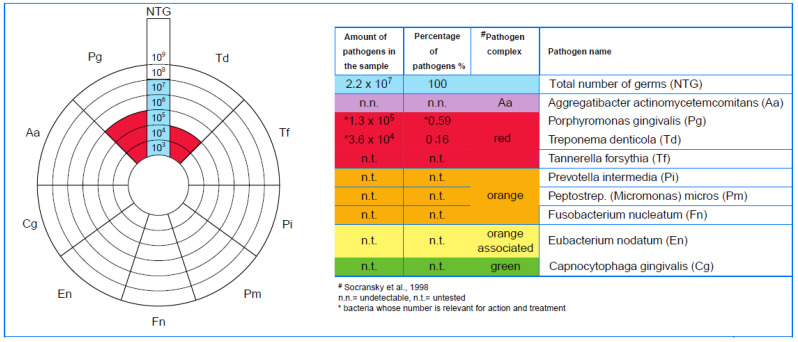
PET-DiagnosticSet: single-site quantitative microbiological analysis results.

As part of evaluating the results, upon the detection of positive DNA for each pathogenic bacterium, the result was reported in colony-forming units (CFUs) quantitatively (Figure 8) and semi-quantitatively. This mode of communication is correlated with the number of germs identified in the sample.

### 2.4. Statistical Analysis

The statistical analysis was performed in the dedicated software SPSS, version 24 (Armonk, NY, USA: IBM). All results are considered significant at a significance level of 0.05; otherwise, the considered level is mentioned.

A *t*-test for paired samples was performed for the comparison of the treatments. The Wilcoxon signed-rank test was carried out when the effects of the two treatments were tested on similar teeth, hence considered in pairs.

## 3. Results

The clinical and microbiological quantification of the results obtained after each applied therapy (SRP and LANAP) allowed for a comparative assessment of their effectiveness.

### 3.1. Clinical Results

For LANAP, improvements were detected in the average evolution of the periodontal pocket depth (PPD), the visible plaque index, and bleeding on the probing index. These three variables showed statistically significant improvements (*p*-value < 0.05). After using LANAP, the periodontal pocket depth decreased on average by 44.4%, the visible plaque index decreased on average by 96.87%, and the bleeding on the probing index decreased on average by 92.85%.

The periodontal pocket depth varied from the mean value on average by 1.98 mm before treatment and by 1.01 mm after treatment, the visible plaque index deviated by 2.28 before treatment and 0.26 after treatment, and the bleeding on the probing index varied by 1.83 before treatment and 0.82 after treatment. For the SRP alone, the depth of the periodontal pockets decreased by 39.5% after treatment, having a standard deviation of 0.97 mm before and 0.75 mm after treatment.

The visible plaque index was reduced by 90% after treatment, with an average deviation from the mean values of 2.34 before treatment to 0.46 after treatment. The bleeding on the probing index decreased after treatment by 88.33%, with an average deviation of 1.58 before and 0.75 after treatment.

Table 1 presents the descriptive statistics for clinical parameters before and after the applied treatments.

**Figure 9:**
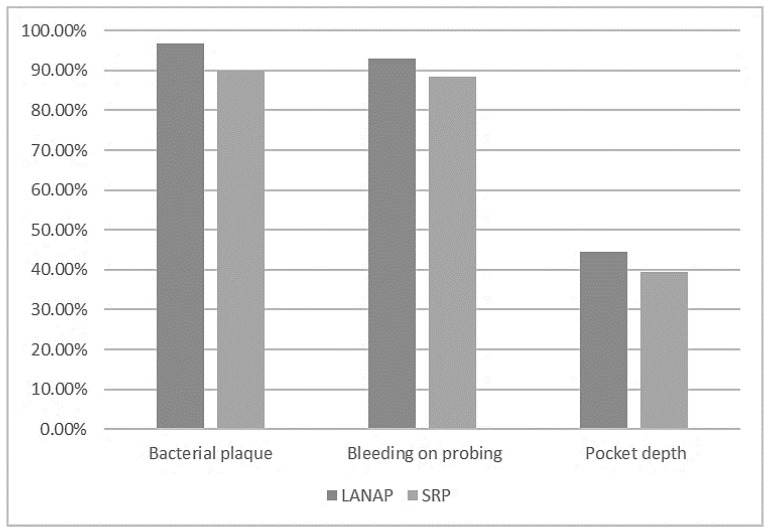
Comparative reduction in dental plaque, bleeding on probing, and periodontal pocket depth parameters after treatment.

Figure 9 shows that although the effects of the two treatments were similar, LANAP had better results than SRP alone.

Table 2 presents the descriptive statistics of the effect of using LANAP or SRP, namely the absolute value of the difference between the variables before and after treatment. These differences were negative in all cases, indicating a decrease in the average periodontal pocket depth, the dental plaque index, and bleeding on the probing index, following the implementation of LANAP and SRP treatments. The effects of the two treatments did not vary statistically (*p*-value > 0.05).

### 3.2. Microbiological Results

For LANAP, the microbiological results showed significant differences in the amount of *Porphyromonas gingivalis* before the treatment and six weeks after the treatment (*p*-value < 0.05). There were no significant disparities regarding the amount of *Aggregatibacter actinomycetemcomitans*, *Treponema denticola*, or the total number of bacteria observed before and six weeks after the treatment.

For SRP, the microbiological results showed no significant disparities at a 0.05 significance level. However, there were significant differences at a significance level of 0.1 for *Porphyromonas gingivalis* before the treatment and six weeks after the treatment. There were no significant differences for the other variables.

Table 3 shows the comparative microbiological results of the assessed microorganisms after LANAP and SRP treatments.

The results showed a more significant reduction in pocket depth and clinical outcomes in the associated therapy (LANAP) compared with the SRP alone, although there were no statistically significant differences between the two therapies, with the exception of the following results:

- The amount of *Porphyromonas gingivalis* (0.05 significance level for LANAP vs. 0.1 for SRP);
- The periodontal pocket depth (44.4% reduction for LANAP vs. 39.5% for SRP);
- The dental plaque index (96.87% reduction for LANAP vs. 90% for SRP);
- The bleeding on the probing index (92.85% reduction for LANAP vs. 88.33% for SRP).

## 4. Discussion

This study provides evidence indicating that LANAP effectively reduces harmful bacteria, namely *Porphyromonas gingivalis,* inside the periodontal pockets, thus improving oral health. This minimally invasive method of treating periodontal disease provides a safe, more comfortable, and effective alternative to conventional periodontal surgery. The procedure effectively reduces periodontal pocket depth, bleeding on probing, and gingival recession, promoting new attachment growth. LANAP aims to reattach the damaged periodontal tissues around the teeth [33].

LANAP is a two-step process that involves first using a specialised laser to discharge the affected gingival tissue and the pathogenic bacteria and then stimulate the development of new, healthy tissue. The laser energy is absorbed by the pigmented bacteria and the affected periodontal tissues, effectively disrupting and destroying the dental biofilm that contains harmful bacteria [37,38,39]. The Nd:YAG laser is then used to stimulate new attachment growth and promote periodontal healing [40,41].

The choice of the therapeutic laser for periodontal treatment (e.g., Nd:YAG laser or diode laser) depends on several factors, such as the type of procedure being performed, the desired clinical outcomes, and the preference of the practitioner [29]. Both types of lasers have advantages and disadvantages, and the ideal choice depends on the specific needs of each case [42]. Nd:YAG lasers have a longer wavelength and are better suited for procedures requiring deeper tissue penetration, such as treating deep periodontal pockets [32]. They better penetrate pigmented tissue, making them appropriate for treating darker skin tones. Diode lasers have shorter wavelengths with a higher absorption in water and haemoglobin [43], thus making them more suitable for patients with lighter skin tones, as they are less likely to cause pigmentation changes [44].

Özberk et al. [45] studied the efficacy of the photobiomodulation treatment (PBMT) associated with SRP as a nonsurgical periodontal treatment in patients affected by type 2 diabetes mellitus. They concluded that clinical attachment level (CAL) improvement occurred. They observed a decrease in pocket depth when the results of PBMT were compared to SRP alone after six months of follow-up. Giannelli et al. [46] revealed that the treatment of periodontal pockets with the Nd:YAG laser can induce the eradication of intracellular and extracellular bacteria of the affected areas and suggested the disinfection with the Nd:YAG laser as a suitable option in the treatment of periodontal disease. Dortaj et al. [29] carried out comparative research between nonsurgical periodontal therapy (NSPT) as monotherapy and NSPT followed by Nd:YAG laser therapy (associated therapy) in stages II-IV periodontal disease. The results showed a more significant reduction in pocket depth in associated therapy compared with NSPT used as monotherapy. However, they considered that the associated therapy did not alleviate the clinical outcomes significantly. Yukna et al. [47] conducted a histological study of periodontal pocket tissues that were collected after applying the Nd:YAG laser beam in LANAP. Their results pointed to the eradication of the affected periodontal tissues and the stimulation of new attachment formation. According to Yukna [48], LANAP is an efficient minimally invasive therapy useful in chronic periodontal conditions. However, longer-term assessment data and controlled trials are necessary to compare full-mouth LANAP treatments with surgical therapies. The systematic search by Jiang et al. [49] showed that laser therapy could enhance the clinical results in short- and medium-term treatments. However, smoking may diminish the adjunct effect of laser therapy. Data obtained by Grzech-Leśniak et al. [50] confirmed that the Nd:YAG laser improved both microbiological and clinical parameters, particularly in moderate and deep periodontal pockets. In a study involving microbial development after using the Nd:YAG laser on fully grown subgingival biofilms, McCawley et al. [41] observed that the use of these laser beams in patients affected by periodontal disease prompted a decrease in the mean proportion (nearly 60%) of the total collected periodontal pathogens.

The standard PCR-PET test (MIP Pharma GmbH, Blieskastel-Niederwürzbach, Germany) uses polymerase chain reaction (PCR) technology to detect specific diseases or conditions. PCR is a highly sensitive and specific method for amplifying and detecting specific DNA sequences, making it a valuable tool for disease diagnosis. PCR-based diagnostic tests offer high sensitivity and specificity. They can also be used to monitor the progression of the disease and the efficacy of the treatments, as well as to screen for potential genetic predisposition to certain diseases [51]. Additionally, it is vital to ensure that the diagnostic tests are performed by qualified laboratory personnel using validated procedures to provide accurate and reliable results [52,53]. The standard PCR-PET tests used in this study were conducted at the MIP Pharma laboratory, with the aim of qualitatively and quantitatively differentiating three periodontal pathogens from the examined samples (*Aggregatibacter actinomycetemcomitans*, *Porphyromonas gingivalis*, and *Treponema denticola*), pathogens that are risk indicators of severe periodontitis.

*P. gingivalis* eludes the host immunity through the mediators of inflammation, harms the periodontal tissues, and augments the threat of systemic disease occurrence associated with periodontitis. The virulence factors of *P. gingivalis* alter the coaggregation, biofilm formation, and dysbiosis specific to the oral microbiota [54,55,56].

This research allowed the assessment of the influence of SRP compared with LANAP to develop a treatment protocol that could enhance the periodontal support of abutment teeth. Thus, the effects of two nonsurgical periodontal therapies, SRP alone and LANAP, on the periodontal healing process were evaluated based on the analysis of their decontamination efficiencyandperiodontal status improvement potential.

Following the quantification of the results, a treatment protocol could be developed to use the SRP and LANAP therapies to improve the periodontal support of abutment teeth. Thus, treating periodontally diseased abutment teeth might become possible, which is a prerequisite for oral rehabilitation using fixed prosthetic restorations for these patients. Oral rehabilitation with fixed prosthetic restorations can have a high social impact by maintaining oral health and increasing the quality of life for the patients, therefore also providing economic impact.

The findings of this study provide solutions to dentists in the current practice, regarding the possibility of recovering the natural teeth and the abutment teeth with periodontal conditions using LANAP. Thus, the study provides insights into oral rehabilitation, periodontology, dental prosthetics, and microbiology.

Further research and careful observation will be necessary for sustaining the clinical and microbiological findings and fully understanding the microbiological impact of LANAP, along with its potential for treating periodontal diseases.

## 5. Conclusions

Within the limitations of this study due to the short time frame of research, the limited number of participants, and the fact that oral hygiene can vary for each individual within the six weeks of the study, we can conclude that even though both LANAP and SRP had similar effects in the alleviation of the microbiological and clinical outcomes of nonsurgical periodontal therapy, patients treated using LANAP had better overall improvements compared with those treated using the SRP alone, with successful impact in reducing the levels of pathogenic bacteria, namely *Porphyromonas gingivalis*, in the studied periodontal pockets, improving the clinical oral health status.

## Figures and Tables

**Table 1:** Clinical parameters before and after LANAP and SRP procedures.

Procedure	Variables	Before Treatment		After Treatment		*p*-Value
		Mean	Std	Mean	Std	
LANAP	Periodontal pocket depth (mm)	6.4286	1.98898	3.5714	1.01635	0
	Visible plaque index	2.2857	1.81568	0.0714	0.26726	0
	Bleeding on probing	4	1.83973	0.2857	0.82542	0
SRP	Periodontal pocket depth (mm)	5.7857	0.97496	3.5	0.75955	0
	Visible plaque index	2.8571	2.34872	0.2857	0.46881	0.001
	Bleeding on probing	4.2857	1.5898	0.5	0.75955	0

**Table 2:** Wilcoxon signed-rank test.

Variables	LANAP		SRP		*p*-Value
	Mean	Std	Mean	Std	
Periodontal pocket depth (mm)	2.8571	1.70326	2.3571	1.27745	0.302
Visible plaque index	2.2143	1.71772	2.5714	2.1018	0.413
Bleeding on probing	3.7143	1.72888	3.7857	1.84718	0.808

**Table 3:** Comparative microbiological results after LANAP and SRP treatments.

Treatment	Variable	Before		After		*p*-Value
		Mean	Std	Mean	Std	
LANAP	*Aggregatibacter actinomycetemcomitans*	58.5714	219.15422	0	0	0.336
	*Porphyromonas gingivalis*	699,385.7143	788,725.1972	50,660	97,481.48353	0.007
	*Treponema denticola*	158,746.1538	142,394.5377	127,672.31	386,171.8167	0.79
	Total number of pathogens	463,607,142.9	261,684,884	302,575,000	787,427,411.5	0.144
SRP	*Aggregatibacter actinomycetemcomitans*	0	0	3538.4615	12,758.10451	0.337
	*Porphyromonas gingivalis*	436,246.1538	568,000.8988	129,896.92	336,900.2959	0.052
	*Treponema denticola*	113,942.8571	30,612.73475	43,185.714	105,652.3898	0.11
	Total number of pathogens	363,428,571.4	651,730,561.9	357,499,286	763,468,672.3	0.984

## Data Availability

The data presented in this study are available on request from the corresponding author. The data are not publicly available due to privacy restrictions.
